# On the Use of Correlation and MI as a Measure of Metabolite—Metabolite Association for Network Differential Connectivity Analysis

**DOI:** 10.3390/metabo10040171

**Published:** 2020-04-24

**Authors:** Sanjeevan Jahagirdar, Edoardo Saccenti

**Affiliations:** Laboratory of Systems and Synthetic Biology, Wageningen University and Research, Stippeneng 4, 6708 WE Wageningen, The Netherlands; sanjeevan.jahagirdar@wur.nl

**Keywords:** biological networks, data simulation, dynamic model, metabolomics, network analysis, nonlinearity, Pearson’s correlation coefficient, permutation test, Spearman’s correlation coefficient, Toeplitz correlation

## Abstract

Metabolite differential connectivity analysis has been successful in investigating potential molecular mechanisms underlying different conditions in biological systems. Correlation and Mutual Information (MI) are two of the most common measures to quantify association and for building metabolite—metabolite association networks and to calculate differential connectivity. In this study, we investigated the performance of correlation and MI to identify significantly differentially connected metabolites. These association measures were compared on (i) 23 publicly available metabolomic data sets and 7 data sets from other fields, (ii) simulated data with known correlation structures, and (iii) data generated using a dynamic metabolic model to simulate real-life observed metabolite concentration profiles. In all cases, we found more differentially connected metabolites when using correlation indices as a measure for association than MI. We also observed that different MI estimation algorithms resulted in difference in performance when applied to data generated using a dynamic model. We concluded that there is no significant benefit in using MI as a replacement for standard Pearson’s or Spearman’s correlation when the application is to quantify and detect differentially connected metabolites.

## 1. Introduction

Metabolite concentration profiles measured in samples, like blood, urine, or tissues and their patterns of variations, are regulated by complex bio-molecular machines. In recent times, there has been a shift towards studying metabolite profiles in a holistic manner by computational and mathematical methods, thanks to the possibility of measuring many metabolites simultaneously using high-throughput techniques like mass spectroscopy (MS) and nuclear magnetic resonance (NMR) [1,2,3].

A biological system can be represented as a complex network of interconnected biomolecular entities [4] which can be visualised in a graphical manner as networks, i.e., sets of nodes that are connected by edges to indicate the existence and the strength of pairwise relationships [5]. This representation shifts the focus towards the relationships among biological entities rather than on their levels; in this light, network and network analysis are fundamental tools from the systems biology toolbox to investigate and understand metabolomic data [6]. When the nodes are metabolites, the network can be called a metabolite-metabolite association network [6,7], and, in modern metabolomic studies, the interest is to reconstruct these associations patterns from observed data measured in well designed experiments.

Association patterns are usually quantified using similarity measures, like correlation and Mutual Information (MI), and most algorithms built for the purpose of network inference make use of one of these two indices [8].

**Figure 1:**
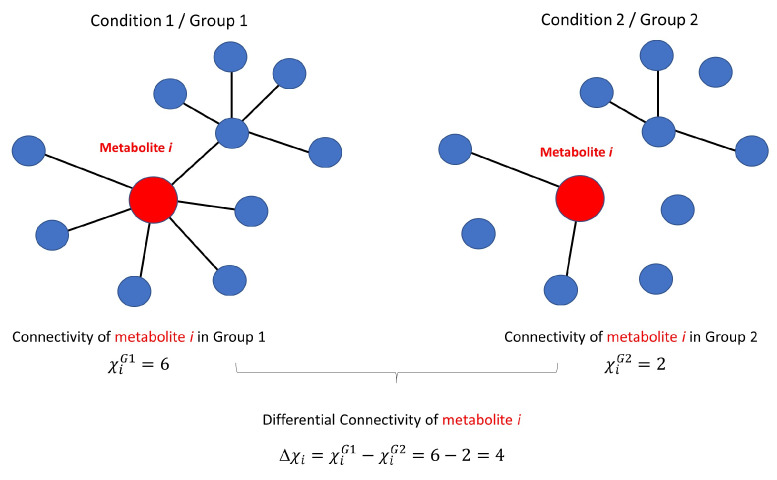
Graphical illustration of the concept of metabolite connectivity and differential connectivity. An ideal unweighted metabolite-metabolite association network involving 10 metabolites is shown under two different conditions. Metabolite *i* is connected with a different number of metabolites (represented by the existence of an edge ) in the two conditions. The connectivity $\chi_{i}$ of metabolite *i* is given by the number of connecting edges (for a generalisation for weighted association networks, see Equation (28) in Section 3.3): 6 under condition 1 and 2 under condition 2. The differential connectivity of metabolite *i* is given by $\Delta \chi_{i}=\chi_{i}^{G1}-\chi_{i}^{G2}=6-2=4$, as described in Equation (29).

Once metabolite-metabolite association networks are reconstructed, they can be analysed in the context of the study design they have been reconstructed, for instance, comparing them across two or more conditions and performing a so-called differential network analysis. In particular, the interest lies in comparing the connections and magnitude thereof for each metabolite between different networks to highlight network differences. The rationale is that, under normal conditions of the system, the metabolites behave in an orchestrated manner and perturbations to the systems, such as those induced by pathophysiological conditions, will induce modifications in the relationships among metabolites that will be reflected in their connectivity patterns. Metabolite connectivity and differential connectivity analysis are illustrated in Figure 1.

In metabolomics, metabolite differential connectivity analysis has been successful to investigate and highlight potential molecular mechanisms underlying cardiovascular diseases [7], age and sex phenotypes [9], acute myocardial events [10], and severe bacterial infections [11]. For instance, Saccenti et al. [7] analysed the metabolite-metabolite association networks specific to different cardiovascular risk patients and reported differential connectivity of Very Low Density Lipoprotein (VLDL) and glucose in high and low risk networks. Azal et al. [11] found the networks specific to patients with necrotising soft tissues infections to be more connected than those of healthy controls and singled out differentially connected metabolites that showed capability of interfering with bacterial biofilm formation.

**Figure 2:**
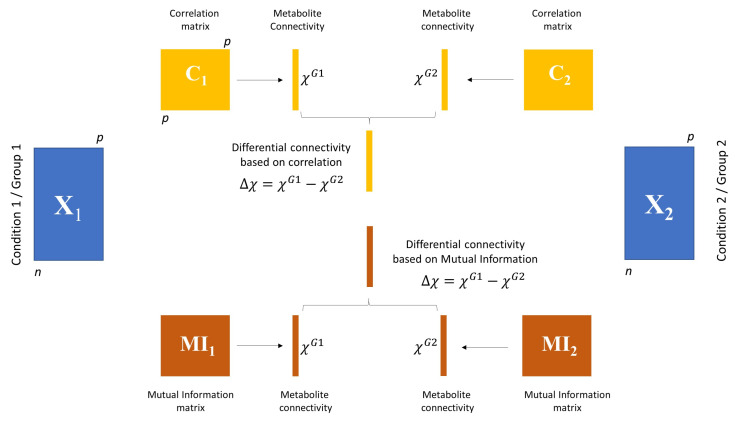
Graphical illustration of differential connectivity analysis. Given two data sets $X_{1}$ and $X_{2}$ of size $n_{1}\times p$ and $n_{2}\times p$ with $n_{1}$ possibly different from $n_{2}$, weighted association matrices are built using either correlation ($C_{1}$ and $C_{2}$, for $X_{1}$ and $X_{2}$, respectively) or Mutual Information (MI) ($MI_{1}$ and $MI_{2}$). Weighted (metabolite) connectivity is then calculated as described in Equation (28) for group 1 and group 2 as $\chi_{i}^{G1}$ and $\chi_{i}^{G2}$. The differential connectivity is given by $\Delta \chi_{i}=\chi_{i}^{G1}-\chi_{i}^{G2}$, and it is calculated using both correlation and MI. Significance is then assessed using a permutation test.

The motivation for this study arose when re-analysing data from Reference [12] in the context of differential analysis of metabolite-metabolite association networks. The original study dealt with the characterisation of metabolites profile associated with sex and age; we were interested in exploring sex-specific patterns of metabolite-metabolite association networks. To this aim, we performed differential network analysis as detailed in the Material and Methods section; briefly, metabolite-metabolite association networks were built starting from the sample correlation matrices or the MI calculated from male and female samples, and a weighted connectivity was calculated as the sum of the (absolute) values of the pairwise Pearson’s correlation (respectively, MI) of a metabolite with every other metabolites, as illustrated in Figure 2. Differential connectivity was defined as the difference between each metabolite connectivity in male and female specific networks, as exemplified in Figure 1. Significance was assessed using a permutation test.

We observed many more differentially connected metabolites when using correlations as a measure of association than with MI. Actually, all 128 measured metabolites showed statistically significant differential connectivity when correlation was used and only 23 when MI was used.

These results were at first surprising: we expected MI to be a more informative measure for quantifying relationship among metabolite than Pearson’s correlations. After all, it is a common place to expect metabolites to exhibit nonlinear behaviour which is better captured by MI. MI (see definitions and equations in Section 3.2) is a non-parametric measure, and it is a comprehensive measure of independence, which makes it superior (in principle) for accounting for both linear and nonlinear dependencies [13]. In fact, Pearson’s correlation can underestimate the dependence between variables when the dependence translates into nonlinear relationships.

**Figure 3:**
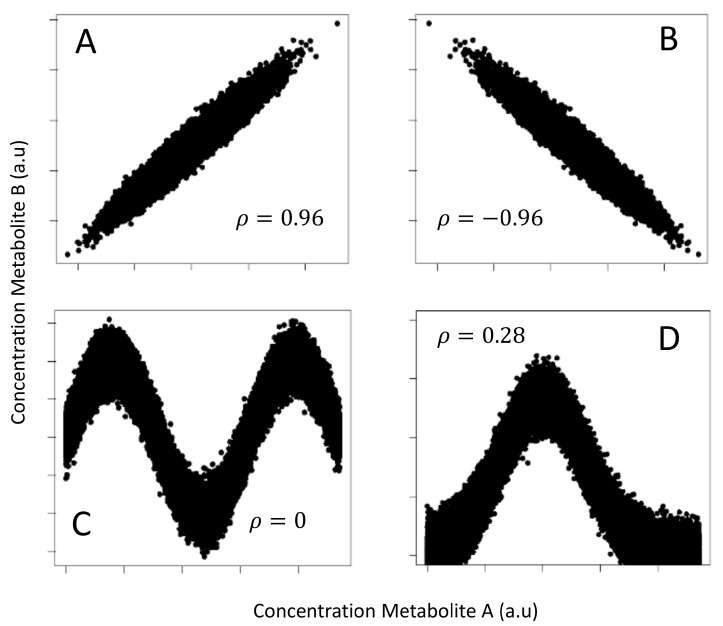
Four different data patterns obtained by plotting the simulated concentration of two metabolites, **A** and **B**, on which Gaussian experimental noise has been added. (**A**) Positive linear relationship, ρ=0.96 (Pearson’s correlation); (**B**) Negative linear relationship, ρ=−0.96; (**C**) Sine-wave relationship, ρ=0 ; (**D**) Bell-shaped relationship, ρ=0.28. In all cases, the MI is 1.32 nats (or 1.90 bits). One nat is the information content of the uniform distribution on the interval [0,e] where *e* is the basis of the natural logarithm. This figure is an adaptation from Table 1 from Reference [13].

An illustrative example is given in Figure 3 that shows four different data patterns (plot of simulated metabolite concentration) all having the same MI (1.32 nats) but very different correlation. Correlation is not able to capture highly nonlinear dependence like in the case shown in panel C, where the metabolites are obviously interdependent.

The question arose of why we observed such counter intuitive behaviour, which led us to explore the question of which association measure is more appropriate for differential analysis of metabolite-metabolite association networks. We started by re-analysing 23 data sets of publicly available metabolomics studies from several research fields, ranging from plant to cancer metabolomics, acquired on different matrices, from cell to tissues, with both MS and NMR. We then compared MI and correlation on simulated data with different correlation structures and properties and using different algorithms to estimate MI (see Section 2.1). Finally, we also compared MI and correlation on simulated data generated using a dynamic model for the NF -κB pathway. In all cases, we found correlation, either of Pearson’s or Spearman’s formulation, to be a more sensitive measure of similarity than MI when used in the context of differential connectivity analysis.

## 2. Results

### 2.1. Differential Connectivity Analysis on Experimental Data

As anticipated in the Introduction, we observed a marked difference when calculating the metabolite differential connectivity (see Equations (28) and (29)) from the metabolite-metabolite association network estimated from blood samples collected from male and females subjects (data set no. 15 in Table 1) [12].

Subsequently, we re-analysed 23 publicly available data sets pertaining metabolomic studies from different fields, from cancer to plant biology. Although different in scope, most studies followed the same simple experimental design: samples were collected from two groups of subjects or from different conditions with the aim of comparing profiles between group 1 and group 2. A list of the data sets considered is given in Table 1, together with a summary of sample size, number of metabolites measured, the experimental platform, and the study design.

For each study, we calculated a weighted adjacency matrix using both Pearson’s correlation and MI via empirical estimation for the two groups, and, for each metabolite, we defined the weighted connectivity, which was compared between the two groups defined by the study design and in which significance was assessed using a permutation test, as illustrated in Figure 2. Results are shown in Table 1. In all cases, the number of differentially connected metabolites (at an α=0.05 confidence level) was much higher when correlation was used as a measure for association and subsequently used to calculate the metabolite connectivity.

This has, of course, tremendous implications for data interpretation. For instance, if differentially connected metabolites are used for enrichment and/or pathway analysis, a great deal of information may be lost. Consider, for instance, data set 12 in Table 1, which collects GC-MS metabolite profiles of healthy men and women. If pathway analysis is performed on the differentially connected metabolites found using correlation or MI, the results are strikingly different: only one pathway (Aminoacyl-tRNA biosynthesis) is found to be enriched (False discovery rate (FDR) < 0.05) when using MI as a measure of association. Eight pathways are found only when using correlation. Results are shown in Table 2. A similar exercise can be performed for data set n. 25 in Table 1. In this case, there is no pathway enriched when using MI.

On the basis of this analysis, we could not draw unequivocal conclusions. In general, there is overlap between the metabolites found to be differentially connected using correlation or MI, but, in many cases, metabolites are found to be differentially connected only when using one of the two measures. For instance, for data set 1 in Table 1, we observed 132 metabolites out of 189 to be differentially connected when using correlation and 90 when using MI, with 64 found with both measures; however, 68 metabolites were found only with correlation and 37 only with MI.

To investigate if these patterns were specific to metabolomic data, we analysed, with the same approach, three transcriptomic data sets, one microbiomic data set, and three data sets pertaining to chemical assays. With the exception of data set 29 and 30, we again observed more differentially connected metabolites when using correlation.

Most data sets are unbalanced, with one group larger than the other: we re-analysed some of the data sets by making them balanced to remove this possible confounding factor. This did not affect the results, which were qualitatively the same: the use of correlation resulted in more differentially connected metabolites also when data is balanced.

### 2.2. Type I Error

Given the results on experimental data, we questioned our validation procedure based on permutation, speculating that the permutation test based on correlation could have resulted, for some reason, in an inflated Type I error, leading to false positives.

To assess this, we devised a simulation strategy where groups 1 and 2 (see Figure 2) were substituted with uncorrelated random data generated under a multivariate normal model, which implies that no variable (metabolite) is differentially connected. Under this simulation scheme, the observed number of differentially connected metabolites should be around 5, i.e., 5% of the total number of metabolites tested, if significance test is performed at α=0.05 level.

**Figure 4:**
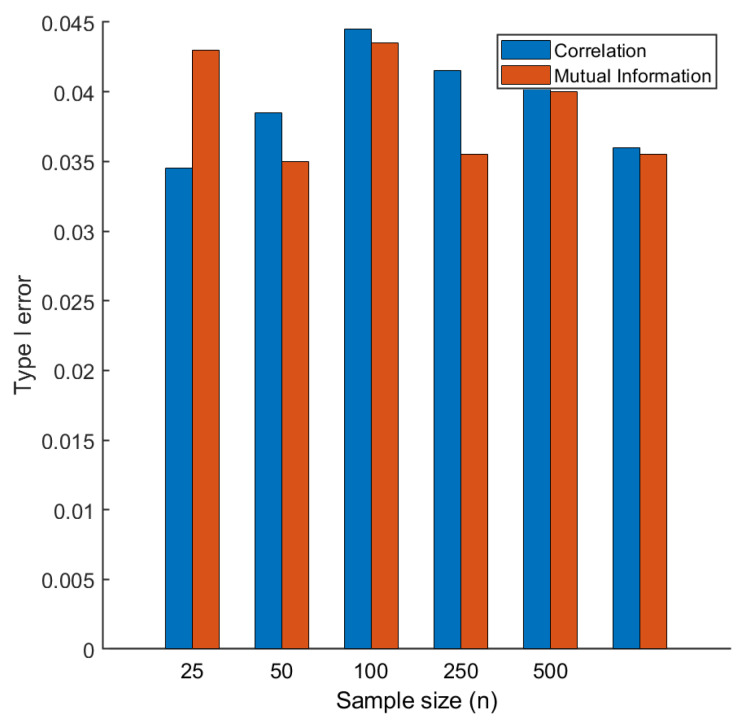
Type I error for the permutation test used to assess the statistical significance of metabolite connectivity. Two data sets $X_{1}$ and $X_{2}$ are generate of size n×20 under an uncorrelated multivariate model ($X_{1}\approx N\left(0,I\right)$. Differential connectivity is calculated as described in Equations (28) and (29) and assessed with a permutation test at the α=0.05 significance level. The overall procedure is repeated 100 times.

We recorded the Type 1 error as a function of sample size *n*, varying *n* from 25 to 500. As shown in Figure 4, the observed Type I error is always around 0.05, independent from the sample size, and from the particular measure of association used. On the basis of this, we could exclude the possibility of inflated Type I error when correlation was used.

### 2.3. Comparison of Correlation and MI on Simulated Data with Known Correlation Structure

We set up a strategy to investigate the behaviour of correlation and MI for differential network analysis further. We generated data with known correlation structures as detailed in Section 3.4.1, Section 3.4.2 and Section 3.4.3 and confronted them with data with uncorrelated structures. The number of variables (i.e., metabolites) was fixed to 20 while the number of samples varied between 10 and 500. In all cases, we varied the strength of the correlation *ρ* between 0 and 1, which means that, apart from the case, ρ=0.

**Figure 5:**
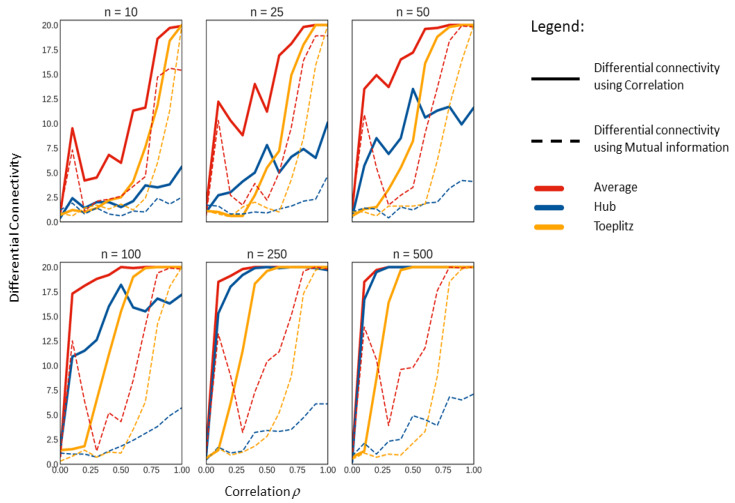
Median of the significant differentially connected variables on all simulated data sets per known correlation *ρ* per sample size *n*.

In this case, we used the four entropy estimators outlined in Section 3.2.1, Section 3.2.2, Section 3.2.3 and Section 3.2.4 to investigate if the particular choice of a method to estimate the entropy necessary to calculate the MI had any effect on the estimation of differential connectivity. Overall, we did not observe any relevant difference when using different methods, and, for this reason, we present and discuss only the results obtained using the empirical probability distribution to estimate the entropy (see Equation (19)). Results are shown in Figure 5.

In all cases, we found more differentially connected metabolites using correlation indices as a measure for association than any of the four MI methods. As it is to be expected, the number of differentially connected metabolites varied with both the sample size and the magnitude of the known correlation *ρ* of the correlation structures. It should be noted that, in our simulation scheme, the differential connectivity is always tested under the alternative hypothesis (see Equation (34)) being true (except when ρ=0); thus, the significant differential connectivity in every situation is expected to be 20 for ρ=0.1 to 1.0 and 0 for ρ=0.

The general trend seen in analysing the number of significantly differentially connected metabolites increases with both sample size *n* and the known correlation *ρ* of the data structures. As for any statistical test, the power of our approach increases with both sample size and effect magnitude. We notice that, at n=500 and ρ>0.8, most methods display the significance of differential connectivity to be 20 with any of the data structures we tested against.

MI is only able to show significant differential connectivity of 20 at ρ>0.8 irrespective of the sample size, indicating a reduction of power to detect differential connectivity. Interestingly, we observed that the performance of MI, in inferring the differential connectivity, drops significantly at ρ=0.3 and then trends upwards again. This observation was consistent for all sample sizes and all methods used to estimate the entropy in this study.

In all cases we observed, the maximal differential connectivity (i.e., 20) is always achieved for smaller values of *ρ* and smaller sample size when using correlation rather than MI.

Given the above mentioned hypothesis, it might be easier to understand why when MI is used as the measure for association; it performs extremely poorly in identifying differential connectivity. The poor performance is unaffected by sample size or by the underlying data correlation structure. These results confirm what was observed when analysing a real life metabolomics data set.

### 2.4. Comparison of Correlation and MI on Simulated Data from a Dynamic Model

The dynamic metabolic model of the NF-κB was used to generate physiologically plausible metabolite concentration profiles for *n* individuals as detailed in Reference [8], mimicking the real life process of data generation from a population of subjects. This data presents metabolites with complex, nonlinear relationships that are almost impossible to simulate with statistical methods; hence this approach gives a better representation of the metabolite-metabolite association patterns observed in real life experimental data.

**Figure 6:**
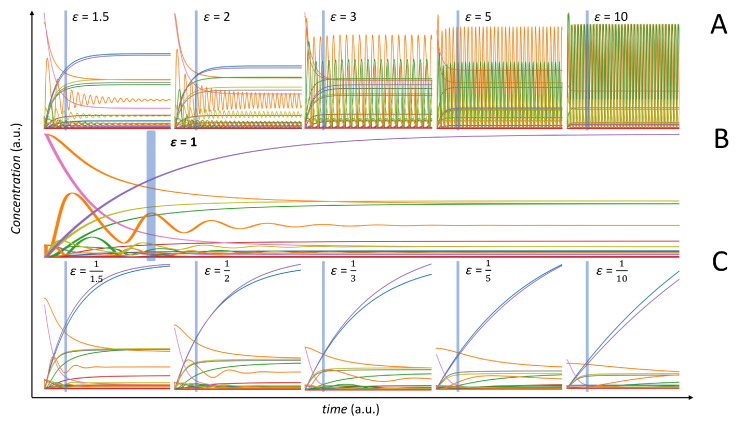
Behaviour of the NF-κB dynamic model. (**A**) Time concentration profiles for model perturbation with ϵ>1. (**B**) Original model. (**C**) Time concentration profiles for model perturbation with ϵ<1. Different colours correspond to different metabolite time profiles. The vertical lines indicate the time sampling point.

Working in a two-groups scenario (see Figure 1 and Figure 2), we varied the kinetic parameters using the multipliers (*ϵ*) to change the behaviour of the entire model. The effect of the modification of the kinetic parameters on the overall model behaviour is shown in Figure 6. Values of ϵ>1 induces fast oscillations in the concentration profiles of certain metabolites (panel A), while values of ϵ<1 flattens out the oscillating behaviour (panel C). Panel B of Figure 6 gives the time concentration profiles for the original, unperturbed, model.

Here, we used *ϵ* as a measure of the perturbation of the dynamic model (data in $X_{1}$), with respect to the original one defined under normal physiological conditions (data in $X_{2}$). However, it should be noted that it is difficult to relate *ϵ* to the number of possibly differentially connected metabolites. This is because it is not possible to predict the relationship among metabolites directly from the structure of the dynamic model. As a matter of fact, the use of the dynamic metabolic model allows a more exhaustive analysis on metabolite associations, but correlations observed in the data do not always reflect the structure of the metabolic network: two metabolites can be direct neighbours in the metabolic network but not correlated; conversely, two metabolites can be very distant in the metabolic network but show high correlation.

The connectivity is formally tested under a null hypothesis scenario, like in the case of data generated under different correlation models (see Section 3.4.1, Section 3.4.2 and Section 3.4.3), but, in this case, the expected connectivity for each metabolite in the NF-κB model for the unperturbed case (ϵ=1) is different from 0.

**Figure 7:**
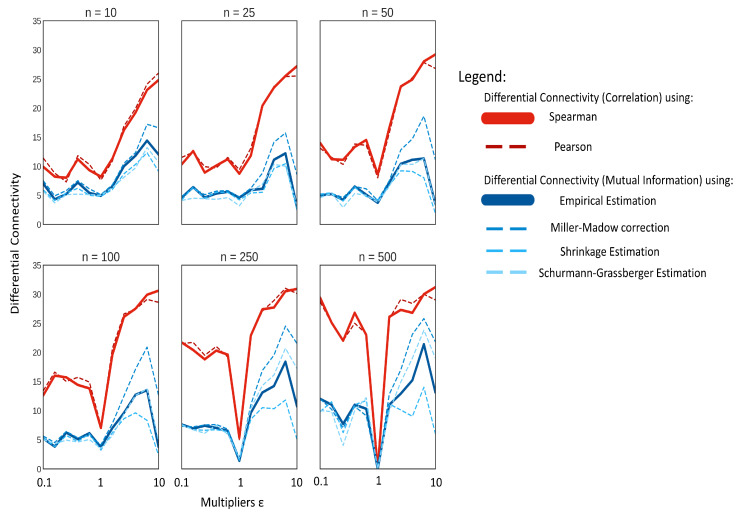
Median of the significant differentially connected variables on data simulated using the NF-κB dynamic model as a function of the model perturbation *ϵ* and the sample size *n*.

In addition, in this case, the use of correlations results, on average, in more differentially connected metabolites, than when using MI, as shown in Figure 7. Pearson’s and Spearman’s correlation performed similarly for most cases, and the marginal difference of Pearson correlation performing better in extremely low sample sizes might be explained by the bias created between the relationship of the two correlation methods, as discussed in Section 2.5.

There is an inherent difference in the change of behaviour in the model with ϵ<1 and ϵ>1, as shown in Figure 6. There is a significant increase in oscillations, at least for some metabolites, when ϵ>1 with the magnitude and the frequency of the oscillations increasing with *ϵ*. This introduces high nonlinearity in the data and may partially explain why MI performs better with ϵ>1 than with ϵ<1. However, this does not explain the differences observed between correlation and MI.

We observed the differential connectivity to be zero for ϵ=1 only for large sample size n=500, suggesting the existence of spurious associations for small sample size and/or instability in the estimation of both correlation and MI.

We speculate that the perturbation in the kinetic parameters may induce pseudo-associations among metabolites that are picked-up by correlation but not by MI, thus increasing metabolites connectivity (see definition in Equation (29)). These pseudo-associations may be stronger when ϵ>1 and the system is oscillating with high frequency, since small changes in kinetics can result in larger variation in concentration when sampling happens at a constant time as in the present case. When ϵ<1, most metabolites exhibit smooth linear and exponential curves, and the variability in concentration is greatly reduced. For example, consider two metabolites, M1 and M2, with the concentration of M1 following an exponential curve for ϵ=1 and ϵ>1, while M2 shows a small oscillation behaviour with ϵ=1 and a large oscillation with ϵ>1. If sampling happens at, say, t=10,000 units, at ϵ=1, there would be small variations in M1 and M2; however, at ϵ>1, there might be large variations in M2 depending on whether the crest or the trough is picked up, especially if the frequency and amplitude are high. This would result in a situation where, when ϵ=1, a small change in M1 is correlated to small change in M2, and, when ϵ>1, a small change in M1 is correlated to a large change in M2; hence, the two variable would show up as differentially connected when the relationship change between them might be less subtle. As the number of samples is increased, the occurrence of such pseudo-associations will be reduced.

In contrast with what was observed with data generated under different correlation models, we observed differences when using different algorithms for the estimation of MI. In particular, the asymptotic bias was large and observable. Indeed, using the Miller-Madow correction (see Section 3.2.2) resulted in a marked increase in performance of MI especially with ϵ>1. On the contrary, the shrinkage estimation of entropy failed to show any increase in performance for inferring differential connectivity as the sample size was increased, confirming previous observations that the shrinkage estimation is more effective at lower sample sizes [42].

When using correlation, for a small sample size (n≤50), the number of differentially connected metabolites for the case of data generated with ϵ<1 seems not to vary, while it increases for ϵ>1. For larger sample size (n≥250) the number of differentially connected metabolites exhibits a symmetric behaviour with respect to ϵ=1. A similar behaviour is observed when using MI, which shows less sensitivity to detect differentially connected metabolites, especially for ϵ<1 and small sample size. The sub-optimal performance of MI to infer connectivity can be explained by considering the analytical relationship existing between Pearson correlation and MI, as shown in Section 2.5.

### 2.5. Relationship between Correlation and MI

In the case of two bivariate variables, $x_{1}$, $x_{2}$, linearly correlated with correlation *ρ*, there is a direct relationship between the MI $MI(x_{1},x_{2})$ and *ρ*. If

(1) $$\left(X_{1},X_{2}\right)\approx N\left(\mu ,\Sigma \right),$$

with

(2) $$\begin{array}{c} \Sigma =\left(\begin{array}{cc} \sigma_{1}^{2} & \rho \sigma_{12}\\ \rho \sigma_{12} & \sigma_{2}^{2} \end{array}\right), \end{array}$$

where $\sigma_{1}^{2}$ and $\sigma_{2}^{2}$ is the variance of $x_{1}$ and $x_{2}$, respectively, and $\sigma_{12}$ their covariance, it holds that (see Equation (2) in Reference [43]):

(3) $$MI\left(X_{1},X_{2}\right)=-\frac{1}{2}\log\left(1-\rho^{2}\right).$$

**Figure 8:**
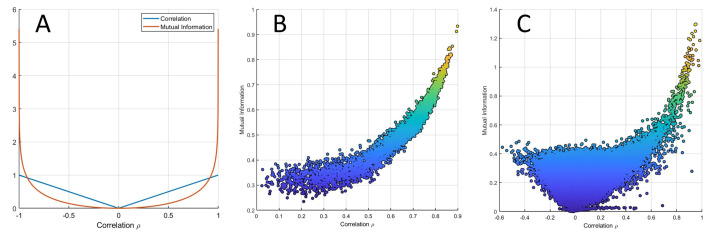
(**A**) MI $MI(X_{1},X_{2})$ of two bivariate variables $X_{1}$, $X_{2}$ linearly correlated with correlation *ρ* as a function of *ρ*. The two curves intersect at approximately ρ=0.916. (**B**) MI versus Pearson’s correlation from data simulated with an average correlation of 0.6 (beta simulation). (**C**) MI versus Pearson’s correlation from experimental data (data set 3 from Table 1).

From Equation (3), it follows that if two variables are linearly (cor)related, their MI is (almost) always smaller than their correlation. This is shown in Figure 8, where the relationship (Equation (3)) is given for −1≤ρ≤: $MI(X_{1},X_{2})$. In particular, it holds that

(4) $$\begin{array}{c} MI\left(X_{1},X_{2}\right)\to \left\{\begin{array}{cc} <\rho & \mathrm{if}|\rho |<0.916\\ =\rho & \mathrm{if}|\rho |=0.916\\ >\rho & \mathrm{if}|\rho |>0.916 \end{array}\right.. \end{array}$$

**Figure 9:**
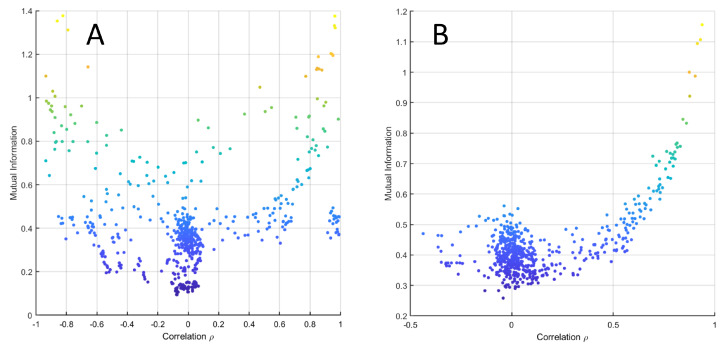
MI versus Pearson’s correlation from data simulated with the NK-kB dynamic model when with (**A**) ϵ=0.1 and (**B**) ϵ=10.

The relationship between MI and correlation is shown for data simulated under the average model (see Equation (38)) in Figure 8B and for experimental data set 3 from Table 1 in Figure 8C, which show good agreement between the analytical relationship between correlation and MI given in Equation (3). Figure 9 shows the same relationship for data generated using the NF-κB dynamic model.

**Figure 10:**
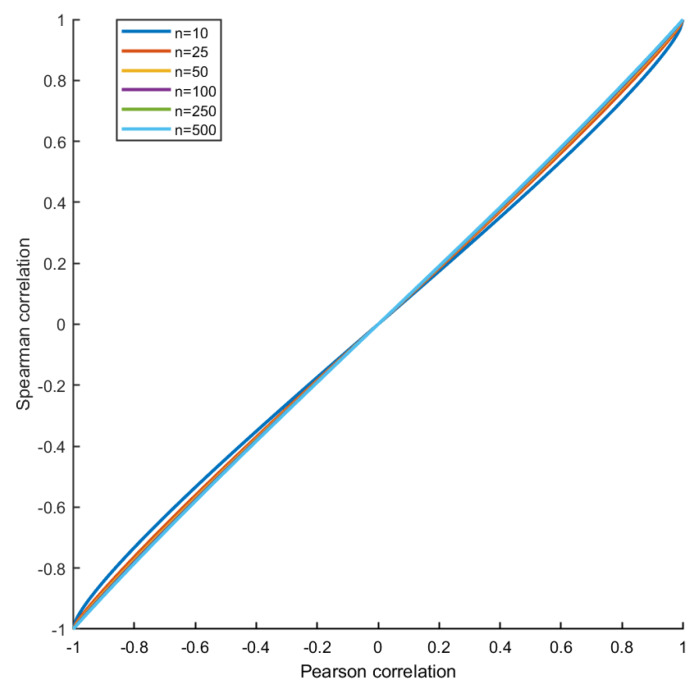
Relationship (see Equation (5)) between the Spearman’s (Equation (7)) and the Pearson’s (Equation (6)) correlation coefficients for linearly correlated data for different sample size *n*.

A similar behaviour is also observed when Spearman’s correlation is used as an index of association. In fact, if there are no ties, the Pearson’s and Spearman’s correlation coefficient are related, for sample size *n*, by the formula [44]

(5) $$\rho_{S}=\frac{6}{\pi (n+1)}\left[\arcsin\rho +\left(n-2\right)\arcsin\left(\frac{\rho }{2}\right)\right],$$

which is shown in Figure 10. For linearly positively correlated variables as in the present simulation, the Spearman’s correlation is biased downwards (in absolute value), and the difference is maximal for ρ=0.577 (respectively, for ρ=−0.577 for negatively correlated variables.). The magnitude of the bias depends on the sample size *n*, but the location where it assumes maximum value is independent from *n*. For a calculation, see Reference [45]. However, for a large sample size (n>50), the bias introduced by taking the Spearman’s correlation in place of the Pearson’s to quantify association is negligible, and, as a consequence, the estimation of the differential connectivity is not affected.

## 3. Materials and Methods

### 3.1. Association Measures

In this study, we used two methods to calculate correlations and four methods to estimate MI as association measures for building the networks.

#### Correlation Indices

The Pearson’s (sample) correlation coefficient [46] between two random variables *X* and *Y* is defined as

(6) $$\rho =\frac{\mathrm{cov}(X,Y)}{S_{X}\times S_{Y}}\;,$$

where $S_{X}$ and $S_{Y}$ is the standard deviation of the measured *X* variables (respectively, *Y*), and cov(X,Y) is the covariance between *X* and *Y*. The Pearson’s correlation coefficient is probably the most used measure of association used in life sciences, and it is a standardised version of the covariance, which, being dependent on the scale of the variables, can vary, in principle, between 0 and +∞ .

The Spearman’s correlation coefficient [47] between two variables, *X* and *Y*, is defined as

(7) $$\rho_{S}=1-\frac{6\sum d_{i}^{2}}{n(n^{2}-1)},$$

where *d* is the difference in rank order between metabolite *X* and *Y*, and *n* is the sample size. The Spearman’s correlation coefficient is an appropriate measure for nonlinear association between two variables, *X* and *Y*.

### 3.2. MI

MI is defined in information theory as the mutual dependence of two random variables *X* and *Y* and can be interpreted as reduction in uncertainty of the outcome of one variable on observation of another variable.

Before defining operatively the concept of MI, we shall introduce the concept of entropy since it is related to MI. Entropy is a measure of the uncertainty about the values that a certain random variable *X*, distributed with probability distribution p(x), can assume.

(8) $$\begin{array}{c} H(X)=-\sum p(x)\log p(x), \end{array}$$

while, if *X* is continuous,

(9) $$\begin{array}{c} H(X)=-\int p(x)\log p(x)dx. \end{array}$$

Equation (8) can be recognised as the the expectation value of −logp(x); thus

(10) $$\begin{array}{c} H(X)=E[-\log p(x)]. \end{array}$$

As an example, assuming a metabolite *X* in which concentration can assume only the values $x_{1}=0.4$, $x_{2}=0.9$, and $x_{3}=1.3$ with probability $p(X=x_{1})=0.2$, $p(X=x_{2})=0.7$, and $p(X=x_{3})=0.1$, the entropy of *X* is

(11) $$\begin{array}{ll} H(X) & =-\sum_{x_{1},x_{2}.x_{3}}p\left(x\right)\log p\left(x\right) \end{array}$$

(12) $$=-\left[0.4\times \log(0.4)+0.7\times \log(0.7)+0.1\times \log(0.1)\right]$$

(13) =0.8018.

The entropy measures the uncertainty of a variable: the higher the entropy, the higher the uncertainty on that variable. Turning to a biological example, if a metabolite shows little variability, i.e., its range of variation is limited, its entropy will also be lower. On the contrary, a metabolite with a large variability will have high entropy. The entropy is usually related to the content of information of a random variable: the higher the entropy, the higher the information content. One can think of a metabolite that does not vary, whatever the experimental circumstances, that assumes value *c* with probability p(X=c)=1; its entropy will be H(X)=0, thus nullifying the information associated to it.

Thus, the calculated entropy of a metabolite will be related to its variance. For instance, if *X* is normally distributed $\approx N(\mu ,\;sigma^{2})$, its entropy is just $\frac{1}{2}\left(\log2\pi \sigma^{2}+1\right)$. The entropy of a variable is maximum when its probability distribution is uniform, and, in contrast with the variance, it can assume negative values.

In practical applications, the probability distribution p(x) is not known a priori but is estimated from the observed distribution of the data, i.e., the empirical entropy is estimated. Estimating entropy is not a trivial task, and many different algorithms exist.

The most common way of expressing the MI between two random variables *X* and *Y* is by expressing the distance between the joint distribution p(X,Y) and product distribution p(X)p(Y) using the Kullback-Leibler divergence [48]:

(14) $$MI\left(X,Y\right)=\sum_{x\in X}\sum_{y\in Y}p\left(x,y\right)\log\left(\frac{p(x,y)}{p(x)p(y)}\right).$$

Since

(15) $$\log\left(\frac{p(x,y)}{p(x)p(y)}\right)=\log\left(\frac{p(x|y)}{p(x)}\right)=\log\left(\frac{p(y|x)}{p(y)}\right),$$

it follows that

(16) MI(X,Y)=H(X)−H(X|Y),

and taking into account the symmetry of information:

(17) H(X)−H(X|Y)=H(Y)−H(Y|X),

an elegant expression of MI as a function of entropy where the MI MI(X,Y) between X and Y, can be obtained as

(18) MI(X,Y)=H(X)+H(Y)−H(X,Y),

where H(X) and H(Y) is the entropy of *X* and *Y*, respectively, and H(X,Y) is the entropy of *X* and *Y*.

Hence, the problem of estimating MI boiled down to the problem of estimating entropy. In this study, we used four different methods to estimate entropy in order to calculate MI, as implemented in the infotheo R package [49].

#### 3.2.1. Entropy of Empirical Probability Distribution

The most common approach to estimate entropy is through the calculation of the probability distribution starting from the empirical data [49]. This is obtained by computing the relative frequency of occurrence of each value:

(19) $${\hat{H}}^{emp}\left(X\right)=-\sum_{x\in X}\frac{\#(x)}{n}\log\frac{\#(x)}{n},$$

where #(x) is the number of data points having value *x*, and *n* is the number of samples.

However, it is necessary to note that empirical estimators are biased downwards and the estimate is always smaller than actual entropy, and the variance of the empirical estimator is dependent on the sample size [50]. More precisely, the variance is upper bounded by $\left(\frac{{\left(\log\;n\right)}^{2}}{n}\right)$.

#### 3.2.2. Miller-Madow Asymptotic Bias Corrected Empirical Estimator

The empirical estimation suffers from an asymptotic bias of $-\frac{|x|-1}{2n}$, where |x| is the number of bins with non-zero probability. This bias can be especially large if the number of bins starts exceeding the sample size. The Miller-Madow correction attempts to get around this problem by adding the asymptotic bias to the empirical estimation of entropy [50]. This correction is given by

(20) $${\hat{H}}^{mm}\left(X\right)={\hat{H}}^{emp}\left(X\right)+\frac{|x|-1}{2n},$$

and it reduces the bias of the estimation without changing the variance.

#### 3.2.3. Shrinkage Estimate of the Entropy of a Dirichlet Probability Distribution

Shrinkage is a popular technique to improve estimators, especially for smaller sample sizes. The shrinkage estimator attempts to combine two estimators in a weighted average with a factor $\lambda^{*}\in \left[0,1\right]$. The two estimators are as follows,

(21) $$\frac{1}{\left|x\right|},$$

(22) $$\frac{\#(x)}{n}.$$

The method shrinks the latter estimate towards the former by minimising the mean square error $\lambda^{*}$. The entropy estimate is then given by

(23) $${\hat{H}}^{shrink}\left(X\right)=-\sum_{x\in X}\hat{p}\lambda^{*}\left(x\right)\log\hat{p}\lambda^{*}\left(x\right),$$

where

(24) $$\hat{p}\lambda^{*}\left(x\right)=\lambda^{*}\frac{1}{\left|x\right|}+\left(1-\lambda^{*}\right)\frac{\#(x)}{n}.$$

The target estimator $\frac{1}{\left|x\right|}$ has low variance and high bias, whereas the unregulated estimator $\frac{\#(x)}{n}$ has large variance and low bias. The benefit of using such a shrinkage method is that the resulting estimator surpasses both of the individual estimates in terms of accuracy and statistical efficiency [51,52].

#### 3.2.4. Schurmann-Grassberger Estimation

The Schurmann-Grassberger method estimates the entropy by utilising a Bayesian parametric strategy assuming samples to be Dirichlet distributed, i.e., multivariate beta distributed given by

(25) $$p\left(X;\theta \right)=\frac{\prod_{i\in \{1,2,...|x|\}}\Gamma \left(\theta_{i}\right)}{\Gamma (\sum_{i\in \{1,2,....|x|\}}\theta_{i})}\prod_{i\in \{1,2,....|x|\}}x_{i}^{\theta_{i}-1}.$$

The entropy of the Dirichlet distribution can be determined by the following with $\theta_{i}=N$ as a constant probability of every event.

(26) $${\hat{H}}^{dir}\left(X\right)=\frac{1}{n+|X|N}\sum_{x\in X}\left(\#\left(x\right)+N\right)(\psi (n+|X|N+1)-\psi \left(\#\left(x\right)+N+1\right)),$$

where, *N* is the prior probability of an event $x_{i}\in X$ assuming that no event $x_{i}$ becomes more probable than another, and ψ(z) as the Digamma function with $\psi \left(z\right)=\frac{dln\Gamma (z)}{dz}$ and Γ(z) as the Gamma function [42,53,54].

It should be remarked that all the estimations used above assume the variables to be discrete in nature; continuous variables are binned before calculations as a pre-processing step. We used the default binning parameters from infotheo R package.

### 3.3. Network Concepts

A network or graph is a graphical representation of the association between objects. In biology, such are molecular components, like genes, proteins, or metabolites, and, in the network, they are represented by nodes. The association between two nodes is represented as link (or edge) connecting the two nodes. The nature of the association among the molecular features can be diverse: in the case of genes regulatory networks, the edges represent regulatory interactions where the protein product of a given gene directly modulates the expression of a target gene; in co-expression networks, the edge represent significant co-expression levels of the connected genes; in protein-protein interaction networks, edges represent the existence of a physical interactions between proteins. In metabolite-metabolite association networks, two metabolite are connected if their concentration levels are significantly correlated.

For manipulation and analysis, networks can be mathematically represented as matrices through the so-called adjacency (also called connectivity) matrix *A*: the rows and columns of the adjacency matrix represent the nodes whereas non-null entries represent links. If the edges are binary indicating only the presence-absence of an association the network is said to be *unweighted*, and the elements $a_{ij}$ of the adjacency matrix describing the association between node *i* and *j* are either 1 or 0:

(27) $$\begin{array}{c} a_{ij}=\left\{\begin{array}{ll} 1 & \mathrm{if}\;\mathrm{there}\;\mathrm{is}\;\mathrm{association}\;\\ 0 & \mathrm{otherwise} \end{array}\right.. \end{array}$$

If the strength of the interaction can be quantified, a weight can be given to the edge; thus, the network is said to be *weighted*: in this case, the elements of a weighted adjacency matrix are real numbers that indicate the strength of the interaction and can vary, for instance, in the [−1, 1] range for correlation, in the [0,+∞) range for MI, or in the [0,1] range for probability.

Each node in a network can be characterised using functions that can be derived from the patterns of its association. A very common measure is the node degree or connectivity, that is, the number of its connection. For a p×p network *A*, the connectivity of the node *i* is given by

(28) $$\begin{array}{c} \chi_{i}=\sum_{j>i}\left|a_{ij}\right|. \end{array}$$

If the network is unweighted, it holds $0<\chi_{i}<p-1$. If the network is weighted, the range of the connectivity depends on the nature of the association measure. If (the absolute value of the) correlation is used, $\chi_{i}$ still ranges between 0 and p−1, in which case, it means that the molecular feature represented by node $a_{i}$ is perfectly correlated with all other nodes in the network. If MI is used, which is in the [0,+∞) range, $\chi_{i}$ also range between 0 and ∞.

#### 3.3.1. Differential Network Analysis

Differential connectivity (see Figure 1 for a graphical overview) is calculated comparing the metabolite connectivity for *p* metabolites measured under two different conditions or in two groups, as exemplified in Figure 2.

Given two data sets $X_{1}$ and $X_{2}$ of size $n_{1}\times p$ and $n_{2}\times p$ with $n_{1}$ possibly different from $n_{2}$, measured under Group 1 (condition 1) and Group 2 (condition 2), respectively (total sample size $n=n_{1}+n_{2}$), and selecting an association measure (either correlation or MI), the differential connectivity $\Delta \chi_{i}$ for the *i*th node (metabolite) is given by

(29) $$\begin{array}{c} \Delta \chi_{i}=\chi_{i}^{G1}-\chi_{i}^{G2}. \end{array}$$

In the simulation study discussed in Section 2.3, data $X_{2}$ is taken to be $\approx N(0,I_{p})$, where $I_{p}$ is the identity matrix of appropriate dimensions. Under this model, the expected connectivity $E[\chi_{i}^{G2}]$ (where E[∗] indicate the expected value of *) is zero, from which it follows that

(30) $$E\left[\Delta \chi_{i}\right]=E\left[\chi_{i}^{G2}-\chi_{i}^{G1}\right]=E\left[\chi_{i}^{G1}\right]=\chi_{i}^{G1}.$$

#### 3.3.2. Permutation Tests to Assess Statistical Significance of Differential Connectivity

The significance of the differential connectivity was assessed implementing a permutation test. First, each and every column of the data matrices $X_{1}$ and $X_{2}$ pertaining to Group 1 and 2 (see Figure 2) is independently permuted; the column values $x_{1},x_{2},...x_{n}$ are replaced by $x_{p(1)},x_{p(1)},...,x_{p(n)}$, where p(1),p(2),...,p(n) are a random permutation of 1,2,...,n. This ensures that the mean and the variance of each column in $X_{1}$ and $X_{2}$ are preserved, but the relationships among the variables are destroyed. For randomised data, the expected metabolite connectivity is $E[\chi_{i}]=0$.

The permuted version of $X_{1}$ and $X_{2}$ are used to build the weighted association matrices, using either correlation or MI, which are then used to compute, for each metabolite, the “permuted” differential connectivity:

(31) $$\begin{array}{c} \Delta \chi_{i}^{perm}=\chi_{i}^{G1,perm}-\chi_{i}^{G2,perm}. \end{array}$$

The permutation procedure is repeated $N_{perm}=10^{3}$ times to build a distribution $D_{i}$ of permuted differential connectivity values for metabolite *i*. This distribution is used to compute the significance of the differential connectivity of metabolite *i*, which is expressed as *P*-value calculated as

(32) $$\begin{array}{c} P_{i}=\frac{1+\mathrm{Num}(D_{i}>\Delta \chi_{i})}{N_{perm}}, \end{array}$$

where $\mathrm{Num}(D_{i}>\Delta \chi_{i})$ indicates the number of elements of $D_{i}$ in which absolute value is larger than $\chi_{i}$, the differential connectivity of metabolite *i* calculated from the original, non-permuted, data $X_{1}$ and $X_{2}$.

This permutation approach is equivalent to a hypothesis testing procedure, where the null hypothesis

(33) $$H_{0}:\Delta \chi_{i}=0$$

is tested against the alternative hypothesis

(34) $$H_{1}:\Delta \chi_{i}>0.$$

### 3.4. Data Simulations

Data were randomly generated under a Gaussian multivariate model with X a n×p data matrix

(35) $$X\approx N(0,\Sigma_{p}),$$

with *n* varying between 10 and 1000.

All variables have been simulated with variance equal to 1, so **Σ** equals the correlation matrix. Three different correlation structures were used as described in the following section.

#### 3.4.1. Toeplitz Correlation Structure

The Toeplitz correlation structure (also called auto-regressive model) describe correlation patterns where adjacent pairs of observations are highly correlated, and those further away are less correlated, with the correlation between the *i*-th and *j*-th observations decay exponentially with respect to |i−j|.

This correlation structure is often used to simulate data in a linear discriminant setting [55], in linear mixed modelling, and in the time series literature as a model for group correlations [56].

The corresponding correlation matrix has the form

(36) $$\begin{array}{c} \Sigma =\left(\begin{array}{cccccc} 1 & \rho & \rho^{2} & \rho^{3} & \cdots & \rho^{p-1}\\ \rho & 1 & \rho & \rho^{2} & \cdots & \rho^{p-2}\\ \rho^{2} & \rho & 1 & \rho & \cdots & \rho^{p-3}\\ \rho^{3} & \rho^{2} & \rho & 1 & \cdots & \rho^{p-4}\\ \vdots & \vdots & \vdots & \vdots & \ddots & \vdots \\ \rho^{p-1} & \rho^{p-2} & \rho^{p-3} & \rho^{p-4} & \cdots & 1 \end{array}\right)\;. \end{array}$$

We generated 10 Toeplitz correlation matrix by varying *ρ* between 0.0 and 1.0 in steps of 0.1.

Given *ρ*, random Toeplitz matrices were generated using the strategy proposed by Hardin and coworkers [56] using the R function simcorTop provided in the supplementary material of Reference [56] and are available at pages.pomona.edu/ jsh04747/research/simcor.r. The parameters used were k=1, ϵ=0.01, and edim = 2. Data matrices were generated using the R function mvrnorm.

#### 3.4.2. Hub Correlation Structure

The hub correlation structure (referred to as hub observation model) describes the situation where *k* groups of variables are presented, and the observations within each group are correlated with a single observation (the so-called hub) with decreasing strength. The *k* groups are independent, i.e., there is no correlation among variables belonging to different groups.

Set the first observation in each group to be the hub-observation, the correlation $\Sigma_{1,i}$ between variable i=1,2,...,g, and the hub-observation

(37) $$\Sigma_{1,i}=\rho -{\left(\frac{i-2}{g-2}\right)}^{\gamma }\left(\rho -\rho_{min}\right).$$

We simulated a hub correlation structure with 2 groups of unequal size (15 and 5, respectively) and varied *ρ* between 0.1 and 1.0 in steps of 0.1 using a quadratic attenuation (γ=2).

Given *ρ*, random hub-correlation matrices were generated using the R function simcor.H provided by Hardin [56]. The parameters used were k=2, ϵ=0.01, γ=2, size = (5,2) and edim = 2. Data matrices were generated using the R function mvrnorm.

#### 3.4.3. Average

Random correlation matrices $\Sigma_{p}$ (with elements $\rho_{ij}$) were generated satisfying the property

(38) $$\frac{2}{p^{2}-p}\sum_{i>j}\left|\rho_{ij}\right|=\rho ,$$

which is the average correlation in $\Sigma_{p}$ is *ρ*, having all variables with a different degree of correlation.

This was accomplished by using the vine method [57,58]. Briefly, correlations are obtained by sampling from a Beta distribution with support −1≤x≤1. The mean *μ* and the variance $\sigma^{2}$ of the Beta distribution are related to the two Beta shape parameters *α* and *β* by the relationships

(39) $$\begin{array}{cc} \mu & =\frac{\alpha }{\alpha +\beta }\\ \sigma^{2} & =\frac{\alpha \beta }{{\left(\alpha +\beta \right)}^{2}\left(\alpha +\beta +1\right)}, \end{array}$$

from which it follows

(40) $$\begin{array}{cc} \alpha & =\frac{1}{\sigma^{2}}-\frac{1}{\mu }\\ \beta & =\alpha (\frac{1}{\mu }-1). \end{array}$$

The mean *μ* was numerically optimised to give average correlation *ρ* between 0.1 and 0.8 in steps of 0.1. The variance $\sigma^{2}$ of the Beta distribution was set to 0.1 in all cases. The corresponding optimised *μ* values were 0.113, 0.116, 0.123, 0.135, 0.163, 0.201, 0.262, and 0.382, respectively, from which the Beta shape parameters *α* and *β* were calculated using Equation (40) and used in the generating vine algorithm (see Section 2.4 in Reference [58]).

### 3.5. Data Generation Using a Dynamic Metabolic Model

To generate data showing correlation patterns similar to those that can be expected in a standard metabolomic experiment used a dynamic kinetic model, we chose a dynamic model describing the lipopolysaccharide-induced activation of Nuclear Factor kappa B signalling pathway (NF-κB, Nuclear Factor kappa-light-chain-enhancer of activated B cells). The model consists of 59 ordinary differential equation describing the reactions involving 35 metabolites. The model describes the intra-cellular signalling pathway that activates NF-κB p65-p50 in response to lipopolysaccharide, which is a gram-negative bacterial endotoxin that triggers an inflammatory response in many cells, including uterine smooth muscle cells. The model was obtained from the BioModels database [59] (www.ebi.ac.uk/biomodels/) with accession number BIOMD0000000489. Full details on the model building and accessory files can be found in the original publication [60].

#### Simulation of Individual Metabolite Concentration Profiles

Subject-specific profiles were generated by varying the $Km_{i}$ and the $k_{i}$ constants for all the 59 reactions and the initial concentrations $c_{m}$ for 4 metabolites with non-zero initial concentrations in the model. The $Km_{i}$ and the $k_{i}$ constants and the initial concentrations $c_{m}$ were sampled from an uniform distribution ≈U(a,b) with lower and upper bounds *a* and *b* set to the reference values ±10% as given in the original publication [60].

For *j*-th individual, the values of *k*, Km, and *c* for any given reaction were defined as

$$k_{i}^{j}\approx U\left(0.9\times k_{i},1.1\times k_{i}\right),$$

$$Km_{i}^{j}\approx U\left(0.9\times Km_{i},1.1\times Km_{i}\right),$$

(41) $$c_{m}^{j}\approx U\left(0.9\times c_{m},1.1\times c_{m}\right).$$

We generated 1000 individual profiles from which we randomly sampled data set of varying size (n=10,25,50,100,250, and 500). In our comparative study, we used these data as data set(s) $X_{2}$, i.e., as a reference data set $X_{2}$ (see Figure 2 for Group (condition) 2).

Data for Group (condition) 1 was constructed by varying the values of $k_{i}^{j}$, $Km_{i}^{j}$, and $c_{m}^{j}$ specific for the *j*-th individual defined in Equation (42) as

$${\tilde{k}}_{i}^{j}=\epsilon \times k_{i}^{j},$$

$${\tilde{Km}}_{i}^{j}=\epsilon \times Km_{i}^{j},$$

(42) $${\tilde{c}}_{m}^{j}=\epsilon \times c_{m}^{j},$$

where *ϵ* is a scaling parameter, equal for all subjects and reactions. We varied *ϵ* over the values $\frac{1}{10}$, $\frac{1}{5}$, $\frac{1}{3}$, $\frac{1}{2}$, $\frac{1}{1.5}$, 1, 1.5, 2, 3, 5, 10 which were used to generate subject specific metabolite profiles as described above. Data was collected in data sets $X_{1}$ of varying size (n=10,25,50,100,250, and 500) and for each *ϵ* value.

### 3.6. Experimental Data

We considered the metabolomic data set compendium compiled by Mendez and coworkers [61]. The compendium contains 10 data sets representative of the three most common metabolomic experimental platforms (nuclear magnetic resonance NMR; gas chromatography mass spectrometry, GC-MS; liquid chromatography mass spectrometry, LC-MS) applied to metabolomic profiling of different biofluids (urine, serum/plasma, faeces). All the data sets pertain case/control studies with a a clear binary outcome available to model (either a primary or secondary outcome of the publication, or a subset of a multi-class study) and have different sample size and number of variables (metabolites) acquired. Data sets characteristics and references are given in Table 1. We made use of the processed cleaned data made accessible via the github link provided in Reference [61] and available in xlsx format. We refer to Reference [61] for more details about the data processing and cleaning. Data were used as provided by Reference [61], with the exception for those data sets where missing data was present: variables with missing data were either removed (data set MTBLS136) or imputed (data set ST001047) using the random forest-based approach implemented in the R package missForest [62].

In addition, we considered other data sets to include also tissues (fat) and plant and fruit extracts together with microbiome data (16S sequencing) and chemical assays on diverse fluids like oil, wine, and coffee. For completeness, we also included two transcriptomic data sets. Data were derived from the original publications or from R packages with which they were distributed, as indicated in Table 1.

The transcriptomic data set were analysed considering only the 250 most and less differential expressed genes between the two classes. Some data sets presented unbalanced groups, and they were analysed retaining the original sample size or making them balanced (see Table 1 for more details).

### 3.7. Software

Calculations were performed using R [63], MATLAB [64], and Python [65]. The R code for differential network analysis is available at www.systemsbiology.nl, under the software tab.

## 4. Discussion

Correlation and MI measures have been widely used in many research applications to quantify and describe the relationships between variables, thus having become the foundations for network inference methods [8]. In general, researchers trained in statistics tend to use correlation based indices, while researchers trained in computer science gravitate towards mutual-information. However, the use of the correlation coefficient is much more widespread in life sciences research than MI: a Pubmed search (March 2020) returned 61,709 hits for “correlation coefficient” against and 3582 hits for “MI”. Inference methods based on correlation can only detect linearly direct associations and can miss nonlinear relations, which play essential roles in many nonlinear systems, such as biological systems [66]. In this light, MI has attractive properties, especially when dealing with the detection of nonlinear relationships [67]. This was one of the main reasons we expected MI to have superior performance in metabolite-metabolite association networks, given the nonlinear nature of the relationships existing among metabolites concentrations. Being based on mutual independence, MI can be considered to be a nonlinear version of correlation that can detect nonlinear correlations (but not direct associations nor dependencies owing to the information of only joint probability) and have the same overestimation problem as correlation [66].

Correlation and MI measure have been compared mostly in the framework of gene networks inferences. Steuer et al. showed an almost one-to-one correspondence between correlation and MI when measuring gene pairwise relationships [68], while Lindolf et al. found no superior merits of MI for constructing co-expression networks [69]. Song et al. examined different correlation-based measure of association and found them to outperform MI in terms of elucidating gene pairwise relationships [70]. In gene ontology studies, it has been observed that, when robust correlation and robust mutual-information has disagreed, the robust correlation findings seemed to be statistically and biologically more plausible [70].

There is little literature on the use of MI in metabolomics applications (12 hits for a Pubmed query “metabolomics AND MI”, performed in March 2020). Numata et al. found that MI was able to detect additional nonlinear correlations undetectable for the Pearson coefficient [71], and Yu et al. concluded that Spearman and MI indexes outperform the other measures to co-associate metabolite and microbiome data [72]. Based on Reference [73,74], Numata et al. also advocated for the use of MI since MI, for pairs of variables, is not altered by homeomorphic (nonlinear) transformations of the data, which may be relevant because metabolomic data rarely yield absolute concentrations, but rather yield ratios of concentrations [75]. However, Saccenti et al. found MI to overestimate chance associations [7]. Correlation are objectively difficult to estimate and are sensitive to experimental noise [76] and to data pre-processing like normalization [77]. However, correlation indexes have nice properties, such as: (i) it can be easily calculated, (ii) it allows for asymptotic statistical tests (regression models, Fisher transformation) for calculating significance, and (iii) the sign of correlation allows one to distinguish between positive and negative relationships.

Although in this study we ignored the directionality of the relationships to build networks and calculate connectivity and perform connectivity analysis, this is a an inherent limitation of MI that cannot capture directionality and changes thereof since it is a strictly semi-positive quantity [78]. In fact, (strong) positive correlation can indicate an equilibrium condition or enzyme dominance, while strong negative correlation can indicate the presence of a conserved moiety [75]. In addition, correlation indices can be calculated with significantly fewer samples than MI [70], and we observed MI to require significantly larger sample sizes to obtain the same robustness attained by correlation. Moreover, the estimation of MI depends on the particular choice of algorithms and user defined parameter setting [79], and we also observed dependence on the estimation algorithm when MI is used for differential connectivity analysis.

On the basis of our investigation concerning the use of correlation and MI for differential connectivity analysis we can conclude that (i) Pearson’s and Spearman’s correlation coefficient are better to detect deferentially connected metabolites than MI methods in metabolite-metabolite association networks created from experimental data, simulated data with known correlated structures, and from a dynamic metabolic model; (ii) when a dynamic metabolic model was used to simulate real-world like observational data, different methods to estimate entropy showed different performance. However, the same could not be concluded when simulated data structures were used. (iii) When analysing the relationship between correlation and mutual-information, we find that mutual-information of two linearly related variables is almost always less than that of their correlation and this was observed in real metabolomics data, simulated data, and data simulated using the NF-κB dynamic model.

Overall, the present investigation indicates that there is no benefit in using MI in place of standard Pearson’s and Spearman’s correlation when the focus of the application is the detection of differentially connected metabolites in differential network analysis.

## Figures and Tables

**Table 1:** *Correlation* and *MI* indicate the number of features found to be statistically significantly differentially connected (at the α=0.05 level using correlation and MI as measure of association). *Only in correlation* and *Only in MI* denote differentially connected features found only using correlation and MI, respectively. *Overlap* indicates those found by both methods. The number of observation (No. observations) is $n=n_{1}+n_{2}$, where $n_{1}$ and $n_{2}$ is the sample size of group 1 and 2, respectively. Study IDs starting with MTBL indicate data available in Metabolights database [14] (www.ebi.ac.uk/metabolights), while those starting with ST indicate data available in the Metabolomics Workbench database [15] (www.metabolomicsworkbench.org). Data set No. 27 was obtained from the RAST database [16] (www.mg-rast.org). Data sets without study ID were derived either from the original publications or from R packages within which they were distributed: BioMark [17], kodama [18], MixOmics [19], and pgmm [20]. Abbreviations: CD, Crohn’s disease; CFS, Cronic fatigue syndrome; E Estrogen; E+P, Estrogen + Progesterone; ES, Ewing sarcoma; IBD, Inflammatory bowel disease; MA, microarray; RMS, Rhabdomyosarcoma; UC, Ulcertive colitis. For data set 24 and 25, the superscripts ‘+’ and ‘−’ indicate the 250 most (the least, respectively) expressed genes, and the superscript *r* indicates a random selecion of 500 genes.

								No. Differentially Connected Features				
No.	Study ID	Ref.	Platform	Type	No. Observations	No. Features	Design	Correlatio	MI	Only in Corr	Only in MI	Overlap
1	MTBLS90	[21]	LC–MS	Plasma	968 (485/483)	189	Sex (M/F)	132	101	68	37	64
2	MTBLS92	[22]	LC–MS	Plasma	253 (142/111)	138	Chemotherapy (before/after)	138	12	126	0	12
3	MTBLS136	[23]	LC–MS	Serum	668 (337/331)	371	Homone (E/E+P)	255	125	167	37	88
4	MTBLS161	[24]	NMR	Serum	59 (34/25)	30	CFS (case/control)	14	12	6	4	8
5	MTBLS404	[25]	LC–MS	Urine	184 (101/83)	120	Sex (M/F)	105	58	51	4	54
6	MTBLS547	[26]	LC–MS	Caecal	97 (46/51)	35	High fat diet (case/control)	35	4	31	0	4
7	ST000369	[27]	GC–MS	Serum	80 (49/31)	181	Adenocarcinoma/Healthy	181	69	112	0	69
8	ST000496	[28]	GC–MS	Saliva	100 (50/50)	69	Debridement (pre/post)	59	31	32	4	27
9	ST001000	[29]	LC–MS	Stool	121 (68/53)	124	IBD (CD/UC)	96	79	33	16	63
10	ST001047	[30]	NMR	Urine	83 (43/40)	149	Gastric cancer/healthy	109	85	42	18	67
11	ST000061		GC-MS	Tissue	118 (59/59)	157	subcutaeus/visceral fat	156	83	73	0	83
12		[31]	NMR	Urine	50 (25/25)	200	cachexia (case/control)	163	57	115	9	48
13		[31]	NMR	Urine	77 (47/30)	63	cachexia (case/control)	63	33	30	0	33
14		[31]	NMR	Urine	60 (30/30)	63	cachexia (case/control)	55	43	15	3	40
15		[12]	GC-MS	Plasma	291(172/119)	128	Sex (M/F)	128	23	105	0	23
16		[12]	GC-MS	Plasma	200 (100/100)	128	Sex (M/F)	103	51	56	4	47
17		[12]	GC-MS	Urine	301 (129/172)	324	Sex (M/F)	256	143	136	23	120
18	MTBLS123	[32]	NMR	Urine	151 (79/72)	63	Shock (pre/post)	63	9	54	0	9
19	ST001243	[33]	GC-MS	Plasma	98 (48/50)	69	Trisomy 21 (yes/no)	69	28	41	0	28
20	MTBLS147	[9]	NMR	Plasma	370 (185/185)	417	Sex (M/F)	417	414	3	0	414
21	KODAMA	[34]	NMR	Urine	80(40/40)	490	Subject (A/B)	459	293	187	21	272
22		[35]	GC-MS	Plant	70 (35/35)	67	Light/Dark	37	19	22	4	15
23	BioMark	[17]	LC–MS	Apple	20 (10/10)	198	Treated/Untreated	124	58	83	17	41
24	MixOmics	[36]	MA	Cell	43 (23/20)^−^	250	Sarcoma (RMS/ES)	250	18	232	0	18
25	MixOmics	[36]	MA	Cell	43 (23/20)^+^	250	Sarcoma (RMS/ES)	250	8	242	0	8
26	MixOmics	[37]	MA	Cell	32 (16/16)^*r*^	500	High/Low dose	405	279	170	44	235
27	4537568.3-776.3	[38]	16S seq	Faeces	145 (71/74)	243	Flock (A/B)	241	150	91	0	150
28	pgmm	[39]	Chemical assay	Oil	50 (25/25)	7	Region (A/B)	4	0	4	0	0
29	pgmm	[40]	Chemical assay	Coffee	43 (36/7)	12	Variety (Arabica/Robusta)	4	11	0	7	4
30	pgmm	[41]	Chemical assay	Wine	130 (59/71)	27	Type (Barolo/Grignolino)	8	10	5	7	3

**Table 2:** Results of pathway enrichment for data set 12 and 25 from Table 1 based on the sets of metabolite found to be differentially connected using correlation or MI as measure of metabolite-metabolite association. FDR: False discovery rate. Empty cells indicate that no metabolite was found to be associated with the given pathway.

	**Pathway Enrichment Based On**			
**Data Set 12**	**Correlation**		**MI**	
**Pathway**	**Raw *P***	**FDR**	**Raw p**	**FDR**
Aminoacyl-tRNA biosynthesis	3 × 10^−12^	3 × 10^−12^	0.0006	0.05
Valine, leucine and isoleucine biosynthesis	3 × 10^−5^	0.001		
Alanine, aspartate and glutamate metabolism	3 × 10^−5^	0.002		
Arginine biosynthesis	0.0004	0.008	0.006	0.18
Glyoxylate and dicarboxylate metabolism	0.001	0.020	0.25	1.00
Glycine, serine and threonine metabolism	0.002	0.020	0.03	0.72
Citrate cycle (TCA cycle)	0.002	0.020		
Phenylalanine metabolism	0.002	0.020	0.09	0.91
Phenylalanine, tyrosine and tryptophan biosynthesis	0.004	0.040		
	**Pathway Enrichment Based On**			
**Data Set 25**	**Correlation**		**MI**	
**Pathway**	**Raw *P***	**FDR**	**Raw p**	**FDR**
Citrate cycle (TCA cycle)	3 × 10^−5^	0.004		
Alanine, aspartate and glutamate metabolism	0.0004	0.016	0.15	1
Glyoxylate and dicarboxylate metabolism	0.001	0.020	0.17	1
Glycine, serine and threonine metabolism	0.001	0.020	0.18	1
Histidine metabolism	0.002	0.036	0.09	1
Tyrosine metabolism	0.004	0.050		

## Abbreviations

The following abbreviations are used in this manuscript:

NF-κB	Nuclear Factor kappa-light-chain-enhancer of activated B cells
NMR	Nuclear magnetic resonance
MI	MI
MS	Mass spectrometry
